# Dietary inclusion of Antarctic krill meal during the finishing feed period improves health and fillet quality of Atlantic salmon (*Salmo salar* L.)

**DOI:** 10.1017/S0007114520001282

**Published:** 2020-04-07

**Authors:** Turid Mørkøre, Helena M. Moreno, Javier Borderías, Thomas Larsson, Hege Hellberg, Bjarne Hatlen, Odd Helge Romarheim, Bente Ruyter, Carlo C. Lazado, Raúl Jiménez-Guerrero, Målfrid T. Bjerke, Tibiabin Benitez-Santana, Aleksei Krasnov

**Affiliations:** 1 Department of Nutrition and Feed Technology, Nofima (Norwegian Institute of Food, Fisheries and Aquaculture Research), NO-9291 Tromsø, Norway; 2 Department of Animal and Aquacultural Sciences, Faculty of Biosciences, Norwegian University of Life Sciences, NO-1432 Ås, Norway; 3 Products Department, Institute of Food Science Technology and Nutrition, ICTAN–CSIC, 28040 Madrid, Spain; 4 Fish Vet Group, Benchmark Norway AS, 0218 Oslo, Norway; 5 Department of Fish Health, Nofima (Norwegian Institute of Food, Fisheries and Aquaculture Research), NO-9291 Tromsø, Norway; 6 Innovation Department, Aker BioMarine AS, NO-1327 Lysaker, Norway

**Keywords:** Aquafeeds, Krill, Health, Metabolism, Connective tissue

## Abstract

There is an urgent need to find alternative feed resources that can further substitute fishmeal in Atlantic salmon diets without compromising health and food quality, in particular during the finishing feeding period when the feed demand is highest and flesh quality effects are most significant. This study investigates efficacy of substituting a isoprotein (35 %) and isolipid (35 %) low fishmeal diet (FM, 15 %) with Antarctic krill meal (KM, 12 %) during 3 months with growing finishing 2·3 kg salmon (quadruplicate sea cages/diet). Final body weight (3·9 (se 0·04) kg) was similar in the dietary groups, but the KM group had more voluminous body shape, leaner hearts and improved fillet integrity, firmness and colour. Ectopic epithelial cells and focal Ca deposits in intestine were only detected in the FM group. Transcriptome profiling by microarray of livers showed dietary effects on several immune genes, and a panel of structural genes were up-regulated in the KM group, including cadherin and connexin. Up-regulation of genes encoding myosin heavy chain proteins was the main finding in skeletal muscle. Morphology examination by scanning electron microscopy and secondary structure by Fourier transform IR spectroscopy revealed more ordered and stable collagen architecture of the KM group. NEFA composition of skeletal muscle indicated altered metabolism of *n*-3, *n*-6 and SFA of the KM group. The results demonstrated that improved health and meat quality in Atlantic salmon fed krill meal were associated with up-regulation of immune genes, proteins defining muscle properties and genes involved in cell contacts and adhesion, altered fatty acid metabolism and fat deposition, and improved gut health and collagen structure.

Wild fish harvested from the ocean and reduced into fishmeal and fish oil are finite resources that are shared across a range of users with increasing demands, from direct human consumption, pig and poultry production and to aquaculture feeds^(1,2)^. As a consequence of high costs of fishmeal and fish oil, traditional small pelagic fish have been increasingly replaced by vegetable feed ingredients in salmon feeds during the past two decades, from 65 to 18 % for fishmeal and from 24 to 11 % for fish oil^(3)^. However, farmed salmon is still a main user of fishmeal and fish oil^(1)^; hence, further complementing feed ingredients from wild fish is required to support continued growth. The average weight of salmon at harvesting is 4–5 kg^(4)^, and it is particularly important to identify feed ingredients that can replace fishmeal and fish oil during the finishing feeding period when the feed demand is highest. It is, however, vital that fishmeal and fish oil replacers not only promote similar growth and survival rates to traditional marine aquafeed ingredients but also ensure that aquatic animal health and welfare is fostered and that the resultant aquaculture products are nutritionally excellent, safe to eat and accepted by the consuming public^(2)^.

Antarctic krill (*Euphausia superba*) is a small marine crustacean organism that has generated strong research interest during the past several years^(5)^. Based on the nutritional profile, krill has been specified with protein, amino acids and ash contents which are comparable to that of fish^(6,7)^. Noteworthy are also the relatively high levels of EPA and DHA^(8)^, phospholipids (PL), vitamins, nucleotides and astaxanthin of krill meal^(9)^. Krill living in the Southern Ocean is considered a sustainable source of protein in aquafeeds^(10)^, and studies with carnivorous fish including Atlantic salmon have documented that krill meal may facilitate efficient reduction in the use of fishmeal in high fishmeal diets^(11–16)^. For example, there are positive effects documented on feed intake and growth at different life stages without compromising fillet quality. However, previous studies have mainly used small fish (<2 kg) and fishmeal as the major dietary protein source. Studies with krill meal supplemented to plant-based/low fishmeal diets, representing the current industrial practice^(3,17)^, and fed to fish reaching normal slaughter weight, are warranted. Furthermore, underlying mechanisms by which krill meal affects fish health and fillet quality are still poorly understood.

As the physical, nutritional and sensory properties of salmon meat at the point of human consumption are influenced by the dietary raw materials^(18,19)^, it is important to monitor and understand the effects on fillet quality when novel feed ingredients such as krill meal are introduced. Thus, improved knowledge is required on the effect of feed resources alternative to forage fish during the finishing period before harvesting when the impact on major salmon flesh quality properties is significant^(20,21)^. For example, the typical pink to deep red colour, fillet firmness and fillet integrity are major quality criteria that dictate the market value of salmon fillets^(22,23)^.

The present study investigates health parameters and meat quality of Atlantic salmon fed a diet where fishmeal was substituted with krill meal during the finishing feeding period with the overarching goal to improve the systemic understanding of dietary krill meal effects on a biochemical, morphological and molecular level.

## Material and methods

### Animal welfare

The experiment was performed according to the guidelines and protocols approved by the European Union (EU Council 86/609; D.L. 27.01.1992, no. 116) and by the National Guidelines for Animal Care and Welfare published by the Norwegian Ministry of Education and Research.

### Experimental design and animal management

The fish used were 800 Atlantic salmon (*Salmo salar* L.; S0 smolt, 16 G, AquaGen) with an initial weight of 2270 g (sd 27 g). The salmon were randomly distributed into eight 125 m^3^ sea cages at The LetSea feed trial unit in Norway, Dønna county in Northern Norway (100 fish in each sea cage) and fed isoprotein (35 %) and isolipid (35 %) 9 mm feed with 15 % fishmeal (FM group) or a diet where fishmeal was partly substituted by 12 % Antarctic krill meal (KM group; Qrill^TM^ Aqua; Aker BioMarine Antarctic AS). The feeds were produced at the Feed Technology Center of Nofima in Titlestad, Norway, formulated to meet known nutritional requirements of salmonid fish^(24)^ and balanced for EPA and DHA content. The FM and KM diets were analysed for moisture (drying at 103°C to stable weight; ISO 6496:1999), crude fat (Soxhlet, with acid hydrolysis; EC 152/2009), crude protein (Nx6.25, Kjeltech Auto System; Tecator), ash (combustion at 550°C, ISO 5984:2002), starch^(25)^, astaxanthin^(26)^ and gross energy (bomb calorimetry; ISO 9831:1998). Additionally, the composition of amino acids (ISO 13903:2005, EU 152/2009; tryptophan EU 152/2009) in the feeds and fatty acid (FA) composition of the feeds and oils^(27)^ were analysed. Formulation and chemical composition of the experimental diets are shown in Table 1. The composition of the fishmeal was: water 8·1 %, protein 69·3 %, lipid 8·5 % and ash 16·6 %. The composition of the krill meal was: water 7·0 %, protein 55 %, lipid 31 % and ash 8 %. The FA composition of the fishmeal, krill meal, fish oil, rapeseed oil, FM and KM diets and fillets is presented in Table 2, and the FA composition of lipid classes and the amino acid composition of the diets are presented in Tables 3 and 4, respectively.

**Table 1.** Formulation and chemical composition of the experimental feeds used in the present study: a low fishmeal diet (FM) and the same diet with fishmeal partly substituted by Antarctic krill meal (KM)* (Percentages)

	FM feed	KM feed
Diet formulation (%)		
LT fishmeal	15	5
Krill meal (Aker Biomarine)	0	12
Wheat (Norgesmøllene)	10	10
Wheat gluten (Tereos)	10	10
Soya protein concentrate (Agrokorn)	14	14
Horse beans (Tereos)	6	6
Soya lecithin (Denofa)	1	1
Maize gluten (Agrokorn)	3	3
Fish oil (Vedde, Norway)	12	8
Rapeseed oil (Emmelev)	21	22
Minerals (Vilomix)	0·6	0·6
Vitamins (Vilomix)	2	2
Monosodium phosphate (Vilomix)	2·6	2·6
Carophyll Pink (10 % Astax) (Vilomix)†	0·05	0·05
Vitamin A (retinol) (IU/g) (Vilomix)	46·4	29·5
Ytrium oxide (VWR)	0·01	0·01
Choline chloride (Vilomix)	0·5	0·5
dl-Methionine (Vilomix)	0·9	0·9
l-Lysine (Vilomix)	1·7	1·7
l-Threonine (Vilomix)	0·4	0·4
Chemical composition (%)		
Crude protein	35·5	35·6
Crude lipids	34·2	34·6
Starch	11·2	11·3
Ash	7·0	6·7
Water	6·1	6·0
Energy (MJ/kg)	24·2	24·6

* The fishmeal, krill meal (Aker BioMarine’s krill fishery) has been certified by the Marine Stewardship Council (MSC) as being sustainable and 100 % traceable.

† Free astaxanthin analysed in diet was 46 and 48 mg/kg in the FM and KM feed, respectively. The krill meal contained <0·2 mg/kg free astaxanthin and 35 mg/kg astaxanthin esters.

**Table 2.** Selected fatty acids (% of total fatty acids) of the fishmeal, krill meal, fish oil, rapeseed oil, FM and KM feed, and fillets of Atlantic salmon fed the different diets† (Mean values, with standard errors for fillet)

	FM	Krill meal	Fish oil	Rapeseed oil	FM feed	KM feed	FM fillet		KM fillet	
							Mean	se	Mean	se
14 : 0	3·2	8·3	7·4	<0·1	2·6	2·6	2·3	0·0	2·4	0·0
16 : 0	12·6	17·1	12·2	4·9	8·2	8·5	9·6	0·1	9·7	0·1
18 : 0	2·0	0·8	1·4	1·5	1·7	1·7	2·3	0·0	2·3	0·0
16 : 1	3·5	3·1	3·9	0·2	1·8	1·7	2·2	0·0	2·2	0·0
18 : 1	13·9	14·4	11·9	66·0	42·3	44·3	41·9	0·1	42·8*	0·1
20 : 1	7·9	1·0	11·6	1·2	6·8	4·8	5·2	0·0	4·8*	0·0
22 : 1	9·4	0·5	18·8	0·2	7·3	5·3	5·0	0·1	4·2*	0·1
18 : 2*n*-6	0·9	1·2	1·3	20·5	14·4	15·1	13·6	0·1	13·8*	0·1
18 : 3*n*-3	0·1	0·1	1·1	9·2	6·0	6·4	5·2	0·0	5·3	0·0
20 : 5*n*-3	6·4	11·2	6·4	<0·1	2·5	2·8	2·3	0·0	2·4*	0·0
22 : 6*n*-3	12·7	8·1	8·5	<0·1	3·5	3·1	3·9	0·0	3·7*	0·1
EPA + DHA	19·1	19·3	14·9	<0·1	6·0	5·9	6·2	0·0	6·1	0·1
Sum *n*-0	17·9	26·2	21·3	7·2	13·4	13·7	14·8	0·1	15·1	0·1
Sum *n*-1	35·9	19·0	46·9	67·7	57·2	56·1	54·8	0·1	54·5*	0·1
Sum *n*-6	1·9	1·6	2·0	20·5	14·8	15·5	15·1	0·1	15·4	0·1
Sum *n*-3	22·5	27·3	20·9	9·2	12·5	12·8	13·0	0·1	13·1	0·1

* Significant difference (*P* < 0·05) between the dietary groups for that parameter.

† A low fishmeal diet (FM), or the same diet with fishmeal partly substituted by krill meal (KM).

**Table 3.** Relative fatty acid composition by lipid class: TAG, mono- and diacylglycerols (MAG), phospholipids (PL) and NEFA of feed given to the Atlantic salmon in the present study* (Percentages of total fatty acids)

	TAG		MAG		PL		NEFA	
	FM	KM	FM	KM	FM	KM	FM	KM
Total	91·2	89·2	2·5	2·4	2·6	5	3·8	3·4
14 : 0	2·7	2·7	3·1	3·2	ND	1·9	2·7	2·1
16 : 0	7·8	7·7	10·2	10·1	20	23·1	14·6	15·1
18 : 0	1·6	1·7	2	1·6	3·1	1·7	2·5	2·4
20 : 0	0·5	0·5	0·6	0·4	0·2	0·1	0·3	0·3
18 : 3*n*-3	5·8	6·2	4·1	4·5	3·4	3·4	3·6	4
20 : 5*n*-3	2	1·6	2	4·4	2·2	13·6	3·9	6·4
22 : 5*n*-3	0·3	0·2	0·7	0·2	0·3	0·4	0·5	0·4
22 : 6*n*-3	2·5	1·9	2·7	3·3	5·4	9·6	7·6	8·6
18 : 2*n*-6	13·3	14·3	13·3	13·6	36·1	20·1	18·2	19·6
20 : 2*n*-6	0·1	0·1	0·2	0·2	0·2	0·1	ND	0·1
20 : 4*n*-6	ND	ND	ND	ND	ND	ND	ND	ND
18 : 1*n*-7	2·7	2·9	2·6	3·4	2	4·5	2·5	3
22 : 1*n*-7	0·2	0·2	0·5	0·3	0·1	0·3	0·4	0·4
16 : 1*n*-9	0·2	0·1	ND	ND	0·1	0·1	0·1	0·1
18 : 1*n*-9	42·2	45·2	36	35·3	16·7	11	23·7	22·6
20 : 1*n*-9	4·6	3·6	4·1	3·1	1·4	0·8	3·2	2·5
24 : 1*n*-9	0·4	0·3	0·5	0·3	1·2	0·3	0·3	0·1
18 : 1*n*-11	0·1	0·2	2·4	0·2	0·1	ND	0·3	0·3
20 : 1*n*-11	1	1	0·8	1·2	0·4	1·8	1·2	1·6
22 : 1*n*-11	6·7	4·8	6·4	4·5	1·3	0·5	4	3
EPA + DHA	4·5	3·4	4·6	7·8	7·6	23·2	11·6	15
Sum *n*–3	11·2	10·2	10	12·9	12	27·5	16·7	20·1
Sum *n*–6	13·7	14·6	14·1	13·9	36·3	20·1	18·3	19·8
Sum *n*–0	13·3	13·2	16·1	15·8	24·2	27·4	21·1	20·5

ND, not detected (less than 0·05 %).

* A low fishmeal diet (FM), or the same diet with fishmeal partly substituted by krill meal (KM).

**Table 4.** Amino acid composition (g/100 g) of the fishmeal (FM) feed and krill meal (KM) feed and of collagen extracted from skeletal muscle of Atlantic salmon fed the experimental feeds (Mean values)

Amino acid/organic acid	FM feed	KM feed	FM collagen*	KM collagen*
Alanine	1·56	1·35	0·07	0·07
Arginine	1·87	1·68	0·03	0·03
Asparagine	2·71	2·46	0·04	0·04
Cysteine + cystine	0·48	0·43	<0·01	<0·01
Glutamic acid (total)	7·14	6·69	0·05	0·06
Glycine	1·54	1·27	0·19	0·20
Histidine	0·70	0·68	0·01	0·01
Hydroxyproline	0·13	0·17	0·04	0·03
Isoleucine	1·32	1·29	0·01	0·01
Leucine	2·56	2·40	0·02	0·02
Lysine	3·03	2·84	0·02	0·02
Methionine	1·47	1·45	0·01	0·01
Ornithin	<0·05	<0·05	ND	ND
Phenylalanine	1·61	1·52	0·01	0·01
Proline	2·26	2·09	0·06	0·06
Serine	1·58	1·46	0·03	0·03
Taurine	0·17	0·13	ND	ND
Threonine	1·54	1·43	0·02	0·02
Tryptophan	0·35	0·36	ND	ND
Tyrosine	1·14	1·09	0·01	0·01
Valine	1·45	1·36	0·02	0·01

* Considering 0·66 % collagen in Atlantic salmon muscle^(41)^. Determinations were performed in triplicate and data correspond to mean values. Standard deviations were in all cases lower than 5 %. Aspartic acid and hydroxylysine were not detected.

Each of the feeds was fed by automatic feeders (S1, Betten) to quadruplicate sea cages during 90 d (September–December 2017), two meals per d. The sea cages were equipped with a feed collecting system (LiftUP, Lift Up AS) to facilitate accurate calculation of the feed consumption and feed conversion ratio, aiming at an overfeeding of 5–10 %. Feed intake per sea cage was registered weekly, as the difference between feed amount and feed spill collected at the bottom of the sea cage, using the feed collecting system. During the period 16 October–23 October, all sea cages were fed Slice^®^ (Skretting), a chemotherapeutant for treating sea lice outbreaks on salmon farms. Before harvesting, the fish were starved for 4 d.

### Slaughter and registrations

All fish within each sea cage were anaesthetised using Finquel vet. (Tricaine Methanesulfonate; Scanvacc) and bulk weighed at the start and termination of the experiment. At the end of the trial, twenty fish were randomly selected from each sea cage for analyses. The first five fish were sampled for blood analyses and subsequent gene expression and histopathological examination. After anaesthesia and blood sampling, the fish were killed by percussive stunning and bled for 20 min in a tank with running seawater (ambient temperature), after sectioning the left gill arch.

**Fig. 1.**
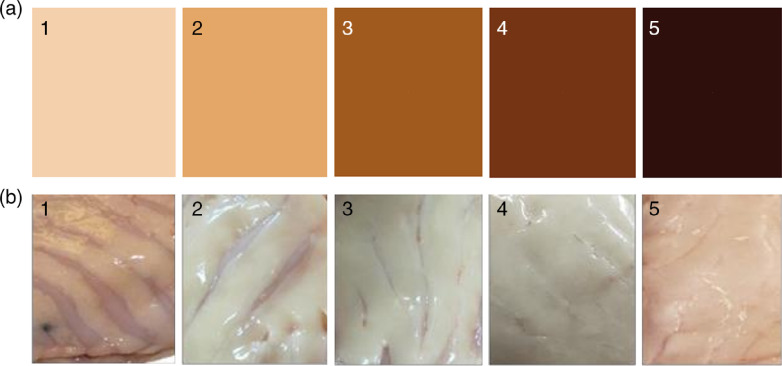
Scale for assessment of visual liver colour (a) and visceral fat according to visibility of pyloric caeca (b) of Atlantic salmon.

Each fish was scored for eye opacity on a scale from 0 to 4, where score 0 represents no visible opacity and score 4 represents opacity of more than 75 % of the cross-sectional area of the lens (complete cataract)^(28)^. The colour of the livers was scored according to a scale from 1 to 5, where score 1 is pale/yellowish and score 5 is dark brown (Fig. 1(a)). Amount of visceral fat (VF) was similarly scored from 1 to 5 according to the visibility of pyloric caeca (PC), where score 1: PC clearly visible, score 2: PC visible, score 3: PC visible as cracks in the VF, score 4: PC visible through the VF, score 5: PC not visible (Fig. 1(b)). VF on the heart surface was scored according to a scale from 0 to 2, where score 0: no VF, score 1: VF on the heart surface, and score 2: severe accumulation of VF on the heart surface. The fish length, sex, body weight before and after evisceration, and weights of livers and hearts were recorded. The fish were filleted by hand, and the weight of right and left fillet was recorded.

### Serum analyses

Blood was collected aseptically from the caudal vein in vacutainers containing clot activator SiO_2_ and centrifuged at 850 ***g*** for 10 min at 4°C. The serum was transferred to separate tubes and frozen at –25°C until analysed spectrophotometrically at Norwegian University of Life Sciences, Oslo Central Laboratory (ADVIA^®^ 1800, Siemens Healthcare Diagnostics Inc.) according to Tietz^(29)^.

### RNA isolation and reverse transcription

Liver, spleen and skeletal muscle were stored in RNAlater (Ambion) before RNA was isolated using an Agencourt^®^ RNAdvance™ Tissue Total RNA Purification Kit (Beckman Coulter Inc.) according to the manufacturer’s protocol. NanoDrop 1000 Spectrophotometer (ThermoFisher Scientific) was used to evaluate the RNA quality and quantity. The quality of the RNA was further assessed with an Agilent^®^ 2100 Bioanalyzer™ RNA 6000 Nano kit (Agilent Technology Inc.). All RNA samples had RNA Integrity Number value higher than 8.

cDNA was synthesised from 1000 ng total RNA in a 20 µl reaction volume using TaqMan Reverse Transcription Reagents (Applied Biosystems, ThermoFisher) with random hexamers as reaction primers. The thermocycling conditions included incubation at 25°C for 10 min followed by 37°C for 30 min and 95°C for 5 min.

### Microarray

Analyses were performed with Nofima’s 44k DNA oligonucleotide microarray Salgeno containing probes to all protein coding genes of Atlantic salmon. Analyses included liver and skeletal muscle (pooled per sea cage). RNA was extracted with a PureLink RNA Mini Kit (Thermo Fisher Scientific). Microarrays were manufactured by Agilent Technologies; the reagents and equipment were purchased from the same provider. RNA amplification and labelling were performed with a One-Color Quick Amp Labelling Kit, and a Gene Expression Hybridization kit was used for fragmentation of labelled RNA. Total RNA input for each reaction was 500 ng. After overnight hybridisation in an oven (17 h, 65°C), arrays were washed with Gene Expression Wash Buffers 1 and 2 and scanned with an Agilent scanner. Subsequent data analyses were performed with Nofima’s bioinformatics pipeline STARS^(30)^. Differentially expressed genes were selected by expression ratio >1·5-fold and *P* < 0·05.

### Histopathology

The following tissues were sampled for histopathological examination: liver (centre), heart (apex), thyroid gland, skin and skeletal muscle (below the dorsal fin, at the lateral line) and hindgut. Tissues were fixed in formalin (4 % formalin, 0·08m sodium phosphate, pH 7·0), processed in a Thermo Scientific Excelsior^®^ tissue processor and embedded in paraffin Histowax using a Tissue – Tek^®^, TEC 5 (Sakura) embedding centre. Embedded tissue was sectioned at 1·5–2 µm using a Leica RM 2255. Microtome sections were mounted on glass slides and stained with haematoxylin–eosin. In addition, gut samples were stained with periodic acid Schiff for mucus and von Kossa’s silver nitrate for Ca. Stained slides were scanned in an Aperio Scan Scope AT Turbo slice scanner and read using Aperio Image Scope (Leica). Slides were examined and scored ‘blind’, that is, without information about the background of the fish. Tissues were examined for pathological lesions and scored on a scale from 0 to 3 where score 0: no pathological changes; score 1: mild changes; score 2: moderate pathological changes; score 3: severe pathological changes.

### Meat quality analyses

The fish were filleted immediately after slaughtering in the pre-rigour state. The fillets were packed individually in sealed plastic bags and stored on ice for 1 week before analysing. Fillet quality analyses included suitability for processing, analysed as degree of fillet gaping (score 0–5) after simulating commercial processing conditions (FHF Industry standard)^(31)^, fillet firmness (instrumentally using a TA-XT2, Stable Micro Systems Ltd and flat-ended 12·5 mm probe at 1 mm/s compression speed)^(32)^, fillet colour (visual assessment, SalmoFan colour score, DSM under standardised light conditions in a Salmon Colour Box, Skretting) and number of melanised muscle segments (myomers). Firmness and colour were analysed above the lateral line just below the cranial part of the dorsal fin and between the caudal part of the dorsal fin and the gut (i.e. Norwegian Quality Cut, NQC^(33)^). The NQC was used for analysing content of protein, fat and astaxanthin, FA composition and collagen characteristics.

### Chemical analyses of fillets and liver

Tissue from forty fish from each dietary group was used for chemical analyses (ten from each sea cage). Protein (Nx6.25 Kjeldahl; NMKL 6) and total astaxanthin content including ester forms were analysed in skeletal muscle (NQC cutlet) (LC-DAD, DSM version 1.5 2009). Total lipids were extracted from fillets (NQC) and liver according to the method described by Folch *et al.*^(34)^, and FA composition was analysed in the chloroform–methanol phase according to the method described by Mason *et al.*^(35)^. In brief, the extracts were dried under N_2_ gas and the residual lipid extract was trans-methylated overnight with 2′,2′-dimethoxypropane, methanolic-HCl and benzene at room temperature. The methyl esters formed were separated in a gas chromatograph (Hewlett Packard 6890; HP) with a split injector, using a SGE BPX70 capillary column (length 60 m; internal diameter 0·25 mm and film thickness 0·25 μm; SGE Analytical Science) flame ionisation detector and HP Chem Station software. The results were analysed using the HP Chem Station software (Hewlett Packard 6890; HP). The carrier gas was He, and injector and detector temperatures were set at 270°C. The oven temperature was raised from 50 to 180°C (10°C/min) and then increased to 240°C (0·7°C/min). The relative amount of each FA was expressed as a percentage of the total amount of FA in the analysed sample.

FA composition of different lipid classes in fillets and feeds was analysed according to Bou *et al.*^(36)^. In brief, NEFA, PL, mono- and diacylglycerols and TAG were separated by TLC using a mixture of petroleum ether, diethyl ether and acetic acid (113:20:2, by vol.) as the mobile phase. The hexane phase was applied onto the TLC plate and dried. The plates were kept in the mixture solution until the liquid reached 1 cm from the upper edge of the plates. The lipids were visualised by dipping the plates in copper sulphate solution. The spots corresponding to NEFA, PL, mono- and diacylglycerols and TAG were identified by comparison with known standards by a Bioscan AR-2000 Radio-TLC & Imaging Scanner and quantified with the WinScan Application version 3.12 (Bioscan Inc.). Lipids were separated on silica gel plates impregnated with silver nitrate (4 % silver nitrate in methanol–water 9:1, v/v) in toluene–ethyl acetate (90:10, v/v), and specific FA were identified by comparison with known standards by a Bioscan AR-2000 Radio-TLC & Imaging Scanner (Bioscan Inc.).

### Connective tissue and collagen determinations

Isolation of connective tissue was determined according to Borderías & Montero^(37)^. Total amino acid analyses of the isolated connective tissue were performed by HPLC using norleucine as an internal standard (Sigma-Aldrich, Inc.) as described by Moreno *et al.*^(38)^. Fourier transform IR spectroscopy analyses were performed by recording IR spectra between 4000 and 650/cm using an IR spectrometer (Spectrum 400, Perkin–Elmer Inc.) equipped with an ATR prism crystal accessory (spectral resolution 4/cm). The microstructure of the collagen fibrils was determined after a consecutive drying process in increased ethanol concentrations (from 30 to 100 % ethanol) and then analysed using a scanning electron microscopy QUANTA 200 (FEI Company). This was operated with low vacuum to allow high-resolution inspection and support the analysis of non-conductive hydrated samples in their original condition with the large-field detector, as it is close to the sample and will prevent loss of electrons. Low vacuum resolution conditions for secondary electrons were 3·0 nm at 30 kV and <12 nm at 3 kV. The accelerating voltage was 23 kV, the low vacuum was 0·50 torr and the working distance was 10 mm. Images of 50 µm were recorded.

### Calculations


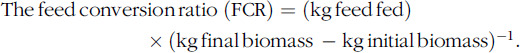


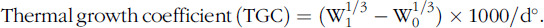

where *W*_0_ is the start weight (g), *W*_1_ is the final weight (g), *t* is the number of days and *d*° is the sum of day degrees.


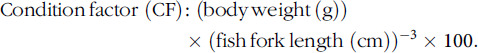


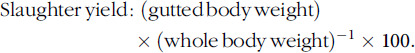


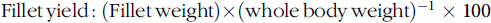


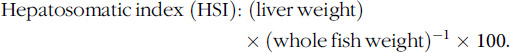


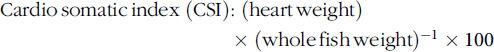


### Statistical analyses

Normality and homogeneity of variance were confirmed, and percentage data were arcsine-transformed before analyses. Nonparametric traits were analysed by Kruskal–Wallis test. Otherwise, the dietary treatments were compared by unpaired *t* test (SAS, version 9.4 for Windows, SAS Institute Inc.). The number of individuals used for fillet quality analyses was based on effect size evaluation on fillet texture measurements by Sigurgisladottir *et al.*^(39)^, based on the method by Nortvedt^(40)^. Sex was not included in the statistical model as the number of males and females was similar for the dietary groups (FM 44 % females and 56 % males; KM 46 % females and 54 % males; *P* = 0·76). The significant difference in the transcript level of the target marker genes between the control and the FM and KM groups was determined by Student’s *t* test for independent samples; the threshold of differential expression in microarray analyses was 1·5-fold. All data are expressed as mean values with their standard errors unless noted otherwise. The level of significance was set at 5 % (*P* < 0·05).

## Results

### Production efficiency and biometric traits

The body weight increased from 2·3 to 3·9 kg during the feeding period. The males were 8·8 % heavier than the females (4·1 *v*. 3·7 kg; *P* < 0·0001), but diets had no effect on final weight, TGC, feed conversion ratio or biometric traits, except for the body shape that was more voluminous of the KM group (higher CF) (Table 5).

**Table 5.** Growth, feed conversion ratio, biometric traits and visual scoring of external and internal traits of Atlantic salmon fed a low fishmeal diet (FM group), or the same diet with fishmeal partly substituted by krill meal (KM group), during the finishing feeding period of 90 d before harvesting* (Mean values with their standard errors)

	FM		KM		*P*
	Mean	se	Mean	se	
Body weight (g)	3833	37	3923	43	0·08
TGC	3·88	0·03	3·92	0·13	0·41
FCR	1·06	0·02	1·07	0·03	0·36
Mortality (%)	1·0	0·4	1·3	0·6	0·72
Biometric traits					
Condition factor	1·41	0·01	1·44	0·01	0·05
Slaughter yield (%)	87·8	0·3	88·0	0·7	0·51
Fillet yield (%)	66·3	0·3	66·9	0·2	0·06
Hepatosomatic index (%)	1·11	0·02	1·12	0·01	0·50
Cardio somatic index (%)	0·14	0·003	0·13	0·003	0·77
Visual scoring					
Wounds	0·4	0·1	0·3	0·1	0·88
Eye lens opacity	0·19	0·11	0·03	0·02	0·09
Visceral fat	3·4	0·1	3·4	0·1	0·68
Fat on heart surface	0·7	0·1	0·4	0·1	0·03
Liver darkness	2·9	0·1	3·7	0·1	<0·0001
Number of dark stained myomera	0·9	0·2	0·5	0·2	0·13

TGC, thermal growth coefficient; FCR, feed conversion ratio.

* Results on body weight, TGC, FCR and mortality are based on registrations of all fish in each of the four sea cages per diet (100 fish per cage initially). For the other traits, *n* 20 randomly selected fish per sea cage (*n* 80 per diet).

### Welfare indicators and organ appearance

While the KM group tended to have lower scores for eye lens opacity (*P* = 0·09), the occurrence of wounds was low and similar for both dietary groups. Accumulation of fat on the hearts was higher in the FM group, and livers were paler compared with the KM group. The score for VF was similar for the FM and KM groups (Table 5).

### Serum analyses

None of the parameters analysed in serum differed significantly between the dietary groups (Table 6). There was an overall negative correlation between serum alanine aminotransferase (ALT) and the visual fat accumulation in the viscera (*r* −0·85; *P* = 0·0007) and on the heart surface (–0·71; *P* = 0·05), while colour, relative size and histopathological changes of the liver showed no significant correlation with the ALT.

**Table 6.** Serum chemistry and histopathology of Atlantic salmon fed a low- fishmeal diet (FM group), or the same diet with fishmeal partly substituted by krill meal (KM group) (Mean values with their standard errors; *n* 4 sea cages/diet, five fish per sea cage)

	FM		KM		*P*
	Mean	se	Mean	se	
Serum chemistry					
Na (mmol/l)	159·3	1·1	160	1·3	0·84
Chloride (mmol/l)	131·2	1·4	132·5	1·4	0·86
Ca (mmol/l)	3·2	0·03	3·1	0·12	0·90
K (mmol/l)	0·6	0·02	0·8	0·16	0·44
Total protein (g/l)	49·3	1·4	48·5	2·2	0·70
Glucose (mmol/l)	5·0	0·03	5·3	0·31	0·10
Creatinine (µmol/l)	15·7	0·5	15·5	0·9	0·81
Alanine aminotransferase (U/l)	12·5	3·5	13·3	2·5	0·74
Histopathological scores (0–3)*					
Heart	0·5	0·1	0·5	0·1	0·76
Spleen	0·0	0·0	0·0	0·0	–
Liver	0·1	0·1	0·4	0·1	0·07
Skeletal muscle	0·1	0·1	0·0	0·0	0·32
Skin	0·0	0·0	0·0	0·0	–
Thyroidea	1·8	0·1	1·8	0·1	0·68
Hindgut	0·7	0·2	0·7	0·2	0·78

* Score 0: no pathological changes; score 1: mild changes; score 2: moderate pathological changes; score 3: severe pathological changes.

### Histopathology

**Fig. 2.**
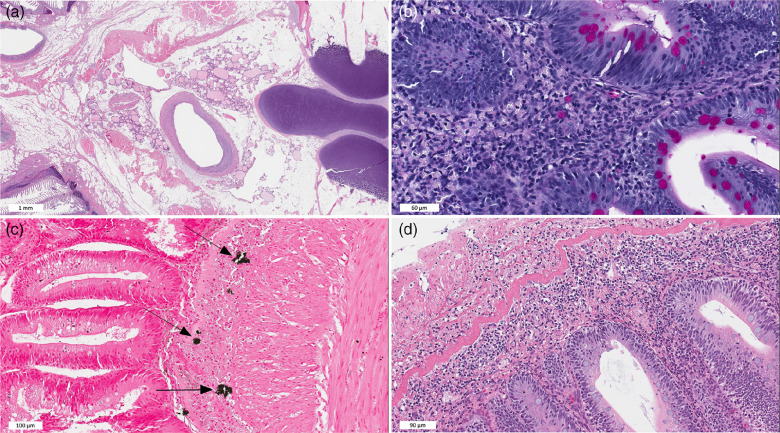
Light micrograph images of (a) thyroid tissue (haematoxylin–eosin (HE) stained), overview; (b) hindgut (periodic acid Schiff stained), mucus stained purple; (c) hindgut (von Kossa stained), focal dystrophic calcinosis; (d) hindgut (HE stained), cell infiltration in *lamina propria* and *submucosa*.

There were no significant differences between the FM and KM groups for any of the histopathological parameters investigated (Table 6). There were no histopathological lesions in skin or spleen. Both groups showed mild lesions in heart and liver, with sparse focal proliferation of epi- and/or endocardium in heart and sparse peribiliar and perivascular inflammatory cell infiltration in liver. One individual in the FM group showed sparse degeneration/necrosis of white skeletal muscle. Both groups showed thyroid lesions, comprising empty follicles with irregular shape and varying epithelial height. Several individuals in both groups had inflammation in the *lamina propria/submucosa* of the hindgut. The overall pathological score of the thyroidea and hindgut was similar for the FM and KM groups. However, periodic acid Schiff staining showed the presence of ectopic epithelial cells containing mucus in *lamina propria* in the hindgut only in salmon belonging to the FM group, and von Kossa staining revealed focal Ca deposits in 20 % of the fish belonging to the FM group, but none of the KM group. Micrographs illustrating examples from the histological examination are shown in Fig. 2.

### Microarray

Analyses of livers showed seventy-three differentially expressed genes whereof forty-four genes had higher expression levels in the KM group (Table 7). Sixteen genes are known or expected to have immune roles, whereof seven and nine immune genes showed, respectively, lower and higher expression in the KM group. Three genes encoding a lectin (*ladderlectin*) were up-regulated in the KM group. Moreover, krill supplementation increased expression of three genes encoding tight junction proteins (connexin and two cadherins).

**Table 7.** Differentially expressed genes (folds), as determined by microarray analyses, in liver of Atlantic salmon fed a low fishmeal diet (FM group), or the same diet with fishmeal partly substituted by krill meal (KM group)* (Mean values)

Gene	KM *v*. FM
*Macrophage colony-stimulating factor 1*	2·0
*Liver-expressed antimicrobial peptide 2*	1·7
*Suppressor of cytokine signalling 2* (two genes)	1·5–1·6
*Ladderlectin-like* (three genes)	1·5–1·8
*Suppressor of cytokine signalling 3b*	−1·7
*CD4-like* (two genes)	−1·7–2·0
*Toll-like receptor 5*	−2·2
*C-C motif chemokine 13* (two genes)	−1·7–2·2
*Complement C1q TNF-related protein*	−2·5
*C1q and TNF-like domains*	−2·5
*Complement component 1q3a*	−3·6
*Cadherin-13-like*	2·4
*Connexin 43·4*	1·6
*Cadherin 2, neuronal*	1·6

* Results are shown as the difference between the KM *v.* FM groups for selected genes where *P* < 0·05 between the dietary groups.

Analyses of skeletal muscle showed 123 DEG whereof 108 genes had higher expression levels in the KM group (Table 8). The largest functional group that responded to feeds was genes for myofiber protein where eleven genes had higher expression in the KM group (seven of them encode different forms of myosin heavy chain) and only one gene with lower expression. Apart from mitochondria (six genes), dietary effects on expression of genes involved a 1·7-fold increased expression of MTP (*Microsomal TAG transfer protein* large subunit minor). The expression differences of immune genes were minor in muscle.

**Table 8.** Differentially expressed genes (folds), as determined by microarray analyses, in skeletal muscle of Atlantic salmon fed a low fishmeal diet (FM group), or the same feed with fishmeal partly substituted by krill meal (KM group)* (Mean values)

Gene	KM *v*. FM
*Suppressor of cytokine signalling*	−1·5
Chromosome maintenance (four genes)	1·6–1·9
Signalling (eight genes)	1·5–1·7
Cell cycle (two genes)	1·5–1·7
Transcription (three genes)	1·7–2·2
Proteasome (two genes)	1·6–2·1
Lysosomes (two cathepsins)	1·7–2·1
Myofiber proteins (twelve genes)	1·5–2·3
Differentiation (four genes)	1·6–2·1
Growth factor (three genes)	1·6–1·8
Mitochondria (six genes)	1·6–1·7
Metabolism lipids (two genes)	1·6–1·7
Metabolism ion (three genes)	1·5–1·8

* Results are shown as difference between the KM *v.* FM groups for selected genes where *P* < 0·05 between the dietary groups.

### Fillet quality

**Fig. 3.**
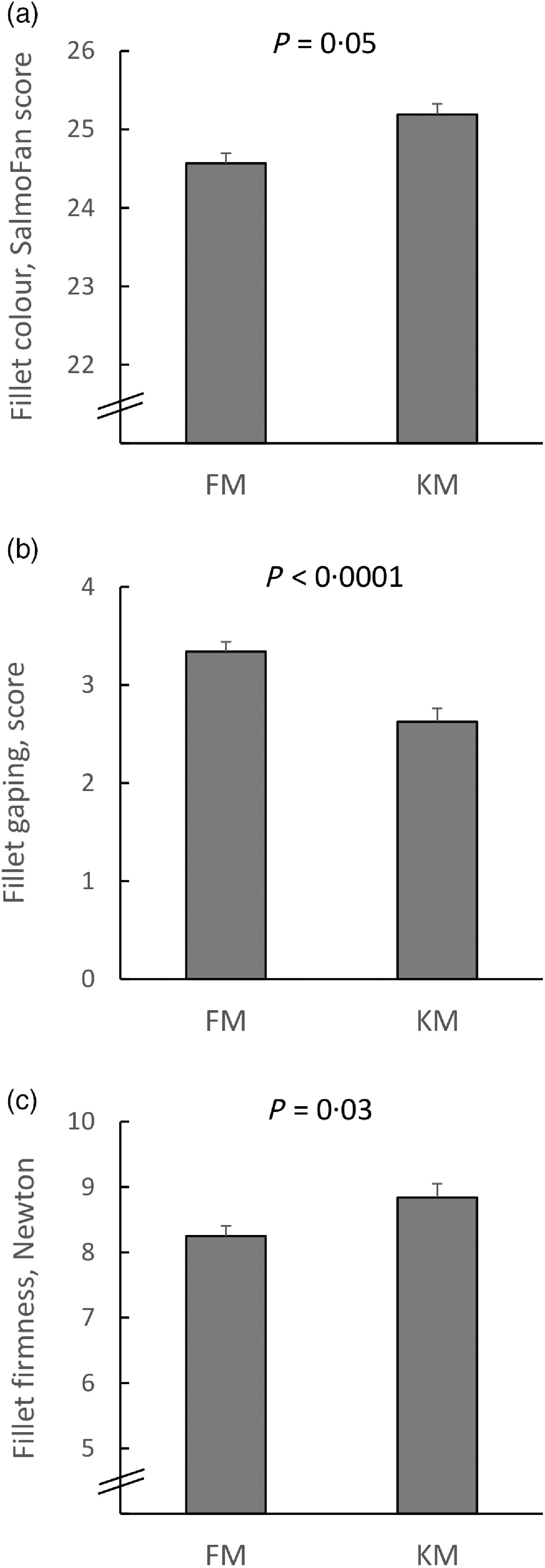
Fillet colour intensity (SalmoFan™) (a), degree of fillet gaping (b) and fillet firmness (c) of Atlantic salmon fed a diet with 15 % fishmeal (FM group), or the same diet with fishmeal partly substituted by krill meal (KM group) during the finishing feeding period of 90 d before harvesting (*n* 80 per diet group; *n* 20 per sea cage).

The KM group had fewer slits between the muscle segments (myomers) in fillets subsequent to handling, and visual fillet colour was significantly redder compared with the FM group. Instrumental analyses revealed that fillets of the KM group were firmer compared with the FM group. Results on fillet gaping, colour and firmness are presented in Fig. 3, where fillet colour and firmness are given as the average of analyses of the anterior and posterior part of the fillet. Fillet colour was less intense in the anterior part (FM: 24·7 (se 0·1) and KM 25·2 (se 0·1); *P* = 0·0002) compared with the posterior part (NQC) (FM: 25·9 (se 0·2) and KM: 26·3 (se 0·1); *P* = 0·11). The astaxanthin level analysed in the NQC was numerically higher in the KM group (7·2 (se 0·2) mg/kg) compared with the FM group (6·6 (se 0·3) mg/kg), but the difference was not statistically significant (*P* = 0·09). Furthermore, the astaxanthin concentration correlated negatively with liver fat content (*r* −0·94; *P* = 0·005). The firmness of the FM group was similar for the anterior and posterior fillet part (8·3 (se 0·2)), while the fillets of the KM group were firmer in the anterior than the posterior region (9·2 (se 0·3) N *v*. 8·5 (se 0·4); *P* = 0·002).

Dietary treatment had no significant effect on the average number of dark hyperpigmented myomers (Table 5), protein content (FM 22·1 (se 0·4) and KM 21·8 (se 0·3); *P* = 0·61) or fat content (FM 21·5 (se 1·3) and KM 21·2 (se 0·6); *P* = 0·94). The fat content in livers was 7·5 (se 0·2) % and 8·6 (se 0·5) % for the KM and FM group, respectively (*P* = 0·18).

### Fatty acid composition of fillets

Salmon fed the KM diet had higher relative content (percentage of total FA) of C18 : 1, C18 : 2*n*-6 and C20 : 5*n*-3 (EPA) and lower relative content of C20 : 1, C22 : 1 and C22 : 6*n*-3 (DHA) compared with the salmon fed the FM diet. The sum of EPA and DHA did not differ significantly between the feed groups. The total content of *n*-1 was lower (mainly due to lower content of 20 : 1*n*-9 and 22 : 1*n*-11), while the sum of *n*-6 FA was higher of the KM group compared with the FM group (Table 2).

The FA compositions of the lipid classes TAG, mono- and diacylglycerols, PL and NEFA in fillets are shown in Table 9. The FA composition of the TAG fraction reflected to a large extent the FA compositions of the diets, with higher percentages of 16 : 0, 18 : 2*n*-6 and a tendency to higher percentage of 20 : 5*n*-3 and lower percentage of 22 : 6*n*-3 in fillet from the KM group relative to the FM group. There were no major differences in the FA compositions of the mono- and diacylglycerols and PL fractions between the two dietary groups. The NEFA fraction that is more available for metabolic conversion than the ester bond lipid fractions was to a larger extent influenced by the diet composition. 20 : 4*n*-3 was reduced and its further desaturation and elongation products in the *n*-3 metabolic pathway, 20 : 5*n*-3, 22 : 5*n*-3and 22 : 6*n*-3, all increased in the KM group compared with the FM group. The same was also shown for the *n*-6 metabolic pathway, with increased percentages of the elongation and desaturation products 20 : 2*n*-6, 20 : 3*n*-6 and 20 : 4*n*-6 in the KM group compared with the FM group. On the other hand, the percentages of the SFA 16 : 0 and 18 : 0 were lower in the KM group relative to the FM group.

**Table 9.** Relative fatty acid composition (% of total fatty acids) of TAG, mono- and diacylglycerols (MAG), phospholipids (PL) and NEFA of skeletal muscle of Atlantic salmon fed a low fishmeal diet (FM group), or the same diet with fishmeal partly substituted by krill meal (KM group) (Mean values with their standard errors)

	TAG				MAG				PL				NEFA			
	FM		KM		FM		KM		FM		KM		FM		KM	
	Mean	se	Mean	se	Mean	se	Mean	se	Mean	se	Mean	se	Mean	se	Mean	se
Total	93·8	0·3	93·5	0·6	1·3	0·4	1·3	0·2	2·8	0·2	2·7	0·3	2·1	0·2	2·5	0·2
14 : 0	2·4	0·01	2·5**	0·01	1·6	0·08	1·5	0·15	1·0	0·28	1·2	0·09	3·1	0·15	2·8	0·11
16 : 0	9·5	0·03	9·7**	0·04	6·0	0·29	6·0	0·62	14·8	0·56	16·1	1·08	16·1	0·95	13·7*	0·39
18 : 0	2·4	0·01	2·5	0·03	2·3	0·60	3·1	0·83	2·7	0·22	2·9	0·22	2·7	0·14	2·3	0·10
20 : 0	0·4	0·00	0·4	0·00	1·2	0·55	1·16	0·37	0·14	0·01	0·05*	0·02	0·3	0·08	0·2	0·01
18 : 3 *n*-3	5·0	0·03	4·9	0·11	4·9	0·61	5·6	0·55	3·8	0·01	3·9	0·28	4·6	0·62	6·3	0·38
20 : 5*n*-3	1·8	0·02	1·9	0·01	3·0	0·29	3·5	0·38	7·2	0·18	7·4	0·49	3·2	0·42	4·7*	0·32
22 : 5*n*-3	0·8	0·00	0·8	0·01	1·3	0·21	1·3	0·10	2·6	0·06	2·3	0·15	0·8	0·12	1·0	0·02
22 : 6*n*-3	2·8	0·01	2·6***	0·02	5·1	1·17	5·3	1·28	24·5	0·62	22·2	1·56	2·6	0·52	3·8	0·38
18 : 2*n*-6	13·4	0·07	13·6*	0·05	11·3	2·31	11·2	2·16	7·2	0·07	6·8	0·34	12·4	1·23	15·3	0·60
20 : 2*n*-6	1·1	0·02	1·1	0·01	0·6	0·13	0·5	0·16	0·7	0·01	0·7	0·07	0·8	0·09	1·1*	0·03
20 : 4*n*-6	0·29	0·00	0·29	0·00	0·58	0·46	1·12	0·49	0·58	0·13	0·66	0·13	0·28	0·04	0·32	0·01
18 : 1*n*-7	1·2	0·06	1·3	0·08	2·0	0·18	2·0	0·23	1·8	0·04	1·8	0·06	2·0	0·26	3·0**	0·06
22 : 1*n*-7	0·7	0·01	0·7	0·01	0·7	0·26	0·8	0·12	0·9	0·14	0·4	0·18	0·9	0·10	1·1	0·02
16 : 1*n*-9	2·1	0·01	2·1	0·03	2·3	0·35	2·2	0·38	1·1	0·06	1·1	0·07	2·0	0·39	1·9	0·24
18 : 1*n*-9	41·9	0·02	42·8***	0·1	31·4	3·53	29·0	4·04	17·5	0·24	16·1	0·71	24·8	1·6	27·9	0·7
20 : 1*n*-9	4·6	0·04	4·2***	0·02	2·0	0·55	2·1	0·32	1·3	0·04	1·1	0·08	2·3	0·23	2·6	0·08
24 : 1*n*-9	0·5	0·00	0·4***	0·00	0·3	0·12	0·2	0·09	1·0	0·06	1·1	0·09	0·5	0·01	0·5	0·02
18 : 1*n*-11	0·2	0·03	0·2	0·02	0·5	0·24	0·5	0·17	0·9	0·20	1·5	0·20	3·0	1·82	0·9	0·36
20 : 1*n*-11	0·7	0·01	0·6	0·03	0·9	0·40	1·3	0·14	0·6	0·00	0·6	0·02	0·9	0·11	1·00	0·05
22 : 1*n*-11	4·1	0·03	3·2***	0·02	1·6	0·25	1·7	0·24	0·8	0·07	0·9	0·14	2·4	0·15	2·0*	0·03
EPA + DHA	4·6	0·03	4·5*	0·03	8·06	1·45	8·7	1·64	31·6	0·74	28·8	2·19	5·84	0·93	8·5	0·70
Sum *n*-3	11·2	0·07	10·8*	0·13	15·2	2·26	16·2	2·07	38·4	0·73	35·6	2·27	12·3	1·79	16·4	1·18
Sum *n*-6	15·2	0·08	15·5*	0·05	13·0	2·04	13·5	1·95	9·5	0·37	9·2	0·18	13·8	1·30	17·4*	0·66
Sum *n*-0	15·2	0·05	15·5**	0·07	12·3	1·93	12·9	2·09	19·8	0·93	21·3	1·43	22·9	1·30	19·6	0·59

* *P* = 0·05, ** *P* = 0·01, *** *P* < 0·001.

### Connective tissue and collagen determinations

**Fig. 4.**
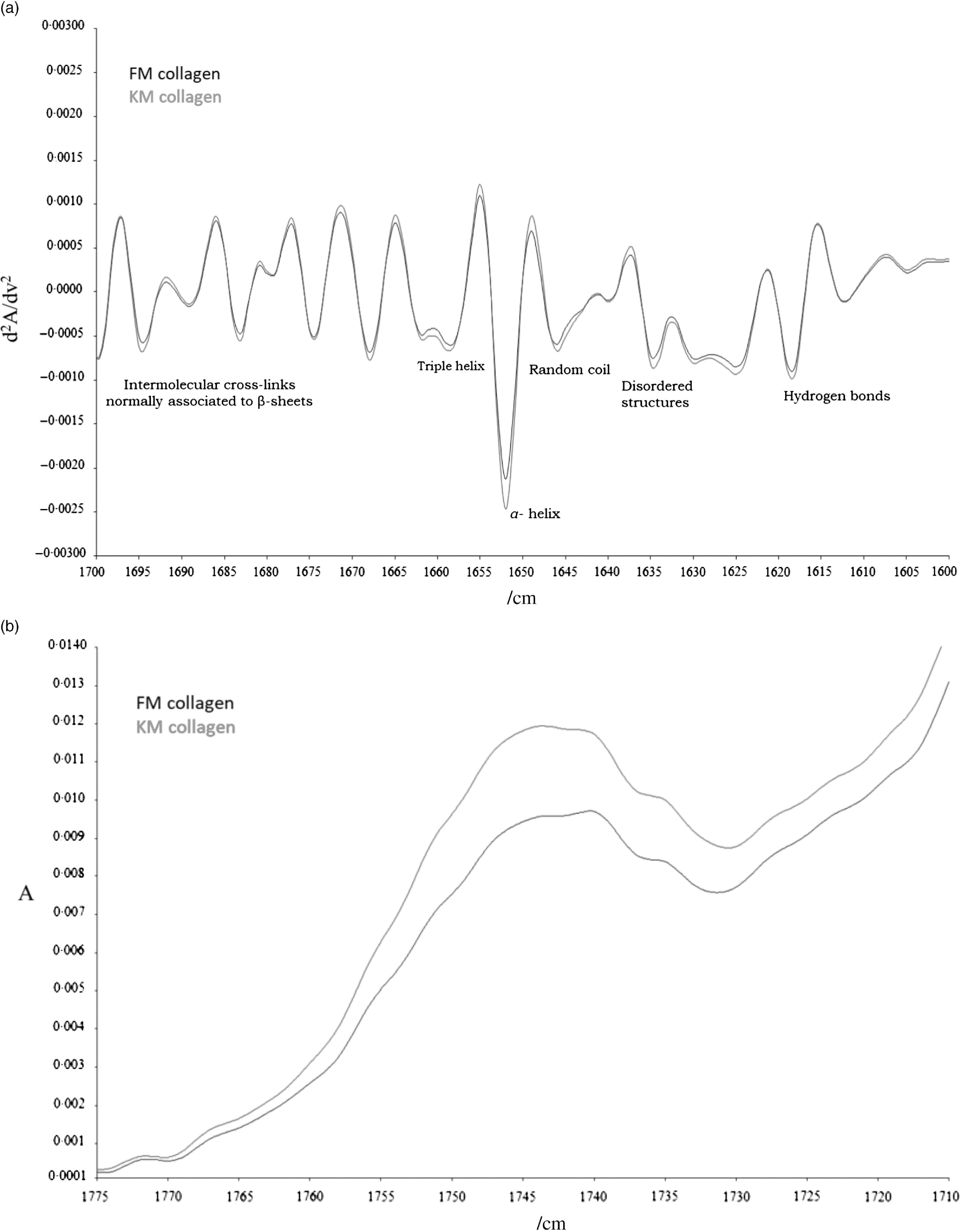
Second derivate of Fourier transform IR spectroscopy (FTIR) spectra of collagen secondary structure (a) and FTIR spectra region 1775–1710/cm for collagen glycolysation (b) of collagen isolated from skeletal muscle of Atlantic salmon fed a diet with 15 % fishmeal (FM group), or the same diet with fishmeal partly substituted by krill meal (KM group) during the finishing feeding period of 90 d before harvesting.

Collagen amino acid composition extracted from salmon skeletal muscle is presented in Table 4. The composition of the collagen molecules was similar for the FM and KM groups. The most abundant amino acids were glycine followed by alanine and proline. In order to study the collagen secondary structure, second derivatives of the amide I band (Fig. 4(a)) were calculated to enhance resolution of the spectral bands and to minimise base-line variations^(42)^. Collagen molecules of the KM group had a lower presence of single *α*-helix (1650/cm), random coil (1645/cm) and disordered structures (1635/cm), as well as lower intermolecular cross-links associated with *β*-sheets (1695–1680/cm). The region of carbohydrate linkage to the collagen molecules (Fig. 4(b)) corresponds to bands appearing in the range of 1775–1710/cm.

**Fig. 5.**
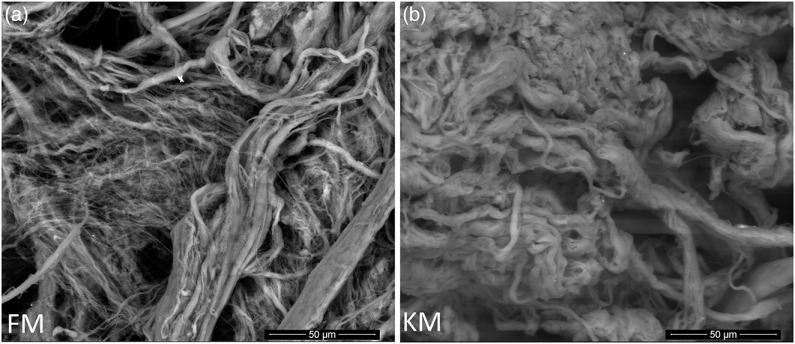
Scanning electron microscope images of collagen fibrils purified from skeletal muscle of Atlantic salmon fed a diet with 15 % fishmeal (FM group), or the same diet with fishmeal partly substituted by krill meal (KM group) (a) during the finishing feeding period of 90 d before harvesting (b).

Scanning electron microscopy analysis of purified collagen confirmed well-defined fibril morphologies (Fig. 5) which could be related to the preserved collagen structure of both collagen as indicated by the Fourier transform IR spectroscopy analysis (Fig. 4(a)). The KM group fibrils had a more compact and well-defined architecture compared with the FM group.

## Discussion

The overall growth rates (TGC 3·9) and feed utilisation (feed conversion ratio 1·07) were above prognosis, as the TGC and biological feed conversion ratio of farmed Norwegian salmon average 2·5^(43)^ and 1·17^(44)^, respectively. Fillet fat content (21 %) was in the upper range of that generally measured in adult salmon (14–20 %)^(45)^. Fast somatic growth and high fat content are results of high energy intake that is typically seen during declining day length in autumn^(20,46–48)^. However, faster growth and, at the same time, lower condition factor and a strong trend towards reduced fat deposition have been seen in salmon fed krill meal; Hatlen *et al.*^(16)^ reported 11 % higher TGC and lower VF in 7 kg salmon fed 10 % dietary krill meal inclusion during 6 weeks. The lack of growth effect by dietary supplementation of krill meal in the present study may be related to the high dietary content of vegetable raw materials, unlike previous studies that used fishmeal as the major protein source. The polar pelagic environment from where the Antarctic krill is harvested is characterised by extreme seasonal changes causing considerable environmental variations and food availability^(49)^. Hence, variation in the effects of krill meal among studies may also be related to krill fishing season.

As the VF was similar for both fish groups, excess VF deposits did not explain the more voluminous body shape of the KM group (higher CF). Rather the 0·6 %-units higher fillet yield (*P* = 0·06) and up-regulation of myofiber proteins indicate stimulated skeletal muscle growth. These results are in line with Hatlen *et al.*^(16)^ who suggested that krill meal may contribute to more efficient utilisation of the feed energy for protein growth (fillet production).

Serum ALT is a widely used index of liver dysfunction, although the reliability is questioned for certain species, such as chicken^(50)^. Krill supplementation did not alter ALT levels or any of the other serum components, as previously reported by Hansen *et al.*^(51)^. Both groups, however, had ALT values that were 1·6 times above the upper limit reported as the normal range for healthy adult salmon of 4–8 U/l^(52)^. Considering the high performance, low mortality rate and low histopathological score of both groups, reference ALT intervals for healthy salmon may need reconsideration. In humans, the presence of obesity increases the risk of elevated liver enzymes by a factor of two to three^(53)^. In the present study, however, an inverse correlation was observed between ALT and viscera and heart fat deposits.

The liver is involved in an array of metabolic and homoeostatic functions, and pale livers have been associated with nutritional disorders^(54)^. The numerically higher fat content in livers of the FM group, together with significantly fattier hearts, may indicate that the PL fraction of the KM diet (5·0 *v*. 2·6 % in the FM diet) influenced the lipid transport and deposition in the fish organs differently than the FM diet. Increased expression of MTP further indicates enhanced lipid transport^(55)^, and the altered FA composition of the free FA in muscle, which are available for metabolism, indicates that krill meal affected the bioconversion of both saturated, *n*-3 and *n*-6 FA. Burri *et al.*^(56)^ reported that in young mice, an equimolar dose of EPA and DHA derived from fish oil modulated fewer pathways (e.g. lipid metabolism and the mitochondrial respiratory chain) than a krill oil-supplemented diet. This suggests that krill-derived *n*-3 PUFA are more bioactive compared with fish oil.

An interesting observation from the liver microarray analyses was a 2·4-fold higher expression of *cadherin-13* (Cdh13) of the KM group. Chd13 is associated with circulating level of the adipocyte-secreted protein adiponectin that has anti-inflammatory potential and plays an important role in metabolic regulation, associated with the fatty liver index in humans^(57)^. The up-regulation of tight junction proteins (connexin, 1·6-fold) indicates improved cell–cell communication of salmon fed krill-supplemented diets, and Willebrords *et al.*^(58)^ reported involvement of connexin hemichannels in non-alcoholic steatohepatitis. Among genes shown to have a role in immune surveillance of KM was up-regulation of *ladderlectin* (three genes, 1·5–1·8 fold) with broad pathogen recognition in rainbow trout^(59–61)^. In addition to liver, dietary krill meal inclusion appeared to improve gut health as ectopic epithelial cells and focal Ca deposits were not observed in KM. Focal accumulation of Ca in necrotic tissue (dystrophic calcification) has previously been observed in intestinal inflammation in Atlantic salmon^(62)^, and the presence of ectopic epithelial cells in the intestine has been associated with chronic feed-induced gut inflammation associated with plant-based ingredients^(63,64)^. The level of plant-based ingredients was similar for the FM and KM diets in the present study. Hence, the observed effects could indicate that krill meal may have anti-inflammatory effects or potentially counteract inflammatory processes in the gut.

Salmon farming is the production of high-quality food. Therefore, it is vital that the fillet quality is in accordance with customers’ expectations. Visual appearance is the most important property of foods in determining their selection^(65)^, while salmon fillets with insufficient firmness are downgraded, leading to severe economic losses to the farming and processing industries^(66)^. The present study was conducted during autumn, which is the most critical period of the year with regard to customer complaints on pale fillet colour, gaping and soft texture, independent of the geographical salmon farming region^(67)^. At the same time, this period is characterised with highest volume of harvested salmon^(4)^.

For salmon fillets, a colour intensity corresponding to SalmoFan colour score of 25 in the posterior standard NQC cutlet meets most customers’ demands, while lower scores increase the risk for quality downgrading^(68)^. Krill meal significantly improved the overall colour, and all fillets of the KM group had SalmoFan score ≥25, while 13 % of the FM group had SalmoFan below the general acceptance level (*P* = 0·03). Astaxanthin is the most common carotenoid used for pigmentation of farmed salmon^(69)^, and pale colour of salmon fillets during periods with high growth has been explained by a negative correlation between feed intake and apparent digestibility of astaxanthin^(26)^. Since the TGC and astaxanthin content in muscle were similar for the FM and KM groups, the improved perceived colour could be related to structural variation in muscle proteins, as was observed by Johnston *et al.*^(70)^. Furthermore, the negative correlation between liver fat content and astaxanthin in skeletal muscle requires further investigation. Dark discoloured spots are major causes to quality downgrading of farmed salmon fillets^(71)^. The KM group had 8 % unit’s lower prevalence of dark spots, but the difference was non-significant.

Improved firmness and integrity (less gaping) by supplementing salmon finishing diets with krill meal showed significant correlation with a range of biochemical and molecular factors, supporting that fillet texture is multifactorial, with complex biological interactions. Previous studies have documented that collagen characteristic is a major determinant of salmon fillet firmness^(38,72)^. In the present study, lower presence of single *α*-helix, lower random coil and lower disordered structures in collagen molecules of the KM group suggest higher preservation of collagen native structure in contrast to the FM group. Also, the collagen of the KM group exhibited lower intermolecular cross-links associated with *β*-sheets that could be related to the lower helix stabilisation by bonds in which Hyp was involved and to the lower presence of hydrogen bonds. As previously reported, all these findings indicate a more preserved native structure and less aggregated collagen molecule in the KM group than in the FM group^(72)^.

The region of carbohydrates linkage to the collagen molecules has been described to be due to –C=O group stretching. As such these may correspond to carbohydrates linked to the collagen molecule by the hydroxyl group reacting with protein carbonyl groups mainly derived from non-enzymatic glycosylation of collagen. The carbohydrates linkage to the collagen molecules has been related to collagen aggregation, mainly at the telopeptide region through the bonds stablished with Arg and Lys amino acids^(73)^. Although salmon from both dietary groups had similar amino acid composition of the collagen molecules, the absorbance in the IR region of carbohydrates was appreciably higher in molecules of the KM group. This would mean increased collagen aggregation at the telopeptide region in comparison with collagen of the FM group. So, it could be stated that the higher native structure preservation of FM collagen molecule is not related to the carbohydrates linked to the telopeptide region. The more aggregated collagen of the FM group would be responsible for the lower ordered fibrils inter-connections and the more compact and well-defined architecture of the KM group in comparison with the FM group. That is, morphology examination by scanning electron microscopy and secondary structure analyses by Fourier transform IR spectroscopy revealed more ordered collagen architecture of the KM group, in addition to lower single *α*-helix, random coil, intermolecular cross-link and more stable cross-links.

Soft flesh of Atlantic salmon has been associated with myocyte detachment and altered extracellular matrix protein distribution^(66)^. Microarray analyses showed that several genes for proteins involved in cell contacts and adhesion were stimulated in muscle and liver of the KM group, but only one gene (*suppressor of cytokine signalling 2*) was bookmarked in both tissues at low level of differential expression; otherwise, responses to feeds in the liver and muscle were completely different. Despite small magnitude of transcriptomic changes, the effects of krill meal supplementation were consistent and characterised mainly by up-regulation of genes as compared with the FM group. The largest functional group that responded to feeds in muscle was genes for myofiber proteins where most of them encode different forms of myosin heavy chain. Moreover, *myomesin-2* that is a gene encoding a protein responsible for the elastic behaviour of the myocyte^(74)^ showed a 1·5-fold increased expression of the KM group compared with the FM group.

### Conclusion

The present experiment aimed to fill key gaps in the current knowledge on how dietary krill meal supplementation affects fish health and meat quality in Atlantic salmon. The results showed that feeding growing and finishing salmon with diets supplemented with krill meal improved fish health and fillet quality that coincided with up-regulation of immune genes, proteins defining muscle properties and genes involved in cell contacts and adhesion, altered FA metabolism and fat deposition, and improved gut health. Higher fillet firmness coincided with more compact and well-organised collagen architecture and predominance of the native collagen structure.
